# Historical ESWT Paradigms Are Overcome: A Narrative Review

**DOI:** 10.1155/2016/3850461

**Published:** 2016-07-17

**Authors:** Heinz Lohrer, Tanja Nauck, Vasileios Korakakis, Nikos Malliaropoulos

**Affiliations:** ^1^European Sportscare Network (ESN), Zentrum für Sportorthopädie, Borsigstrasse 2, 65205 Wiesbaden-Nordenstadt, Germany; ^2^Institute for Sport and Sport Sciences, Albert-Ludwigs-Universität Freiburg im Breisgau, Schwarzwaldstraße 175, 79117 Freiburg, Germany; ^3^European SportsCare, 68 Harley Street, London W1G 7HE, UK; ^4^Aspetar Orthopaedic and Sports Medicine Hospital, Sport City Street, P.O. Box 29222, Doha, Qatar; ^5^Institute for Postgraduate Studies in Manual Therapy, 111528 Athens, Greece; ^6^Thessaloniki Sports and Exercise Medicine Clinic, Asklipiou 17, 54639 Thessaloniki, Greece; ^7^National Track and Field Centre, Sports Medicine Clinic of S.E.G.A.S., Kautatzoglion Stadion, Agiou Dimitriou 100, 54636 Thessaloniki, Greece; ^8^Sports Clinic, Rheumatology Department, Barts Health NHS Trust, Bancroft Road, London E1 4DG, UK; ^9^Centre for Sports and Exercise Medicine, Queen Mary, University of London, Bancroft Road, London E1 4DG, UK

## Abstract

Extracorporeal Shock Wave Therapy (ESWT) is a conservative treatment modality with still growing interest in musculoskeletal disorders. This narrative review aims to present an overview covering 20-year development in the field of musculoskeletal ESWT. Eight historical paradigms have been identified and put under question from a current perspective: energy intensity, focus size, anesthesia, imaging, growth plates, acuteness, calcifications, and number of sessions. All paradigms as set in a historical consensus meeting in 1995 are to be revised. First, modern musculoskeletal ESWT is divided into focused and radial technology and the physical differences are about 100-fold with respect to the applied energy. Most lesions to be treated are easy to reach and clinical focusing plays a major role today. Lesion size is no longer a matter of concern. With the exception of nonunion fractures full, regional, or even local anesthesia is not helpful in musculoskeletal indications. Juvenile patients can also effectively be treated without risk of epiphyseal damage. Further research is needed to answer the question about if and which acute injuries can be managed effectively. Treatment parameters like the number of sessions are still relying on empirical data and have to be further elucidated.

## 1. Introduction

Explosive events in nature (e.g., lightning stroke) and technics (e.g., airplanes breaking through the sound barrier) create shock waves. In principle, these acoustic waves transmit energy “from the point of generation to remote regions.” The principle of this natural phenomenon has been transferred to medical application. “Shock and pressure waves are pulses, while ultrasound is a continuous oscillation” [1]. Shockwaves are generated extracorporeally (electrohydraulic, piezoelectric, or electromagnetic). The resulting energy is focused by concentrating reflectors and is noninvasively transmitted inside the body to induce therapeutic effects at a target area. So-called radial shockwaves have different physical characteristics. They are pressure waves and not real shockwaves. Different tissues possess different acoustic impedance. At the interface between these tissues, acoustic energy is released and transformed into mechanical energy [1].

Starting in 1980, extracorporeal shockwaves were applied transcutaneously for the first time in medicine to destroy a kidney stone in a human [2]. Since then, several million people have benefited from this noninvasive method. As a result of the high energy applied in Extracorporeal Shock Wave Lithotripsy, much research has been performed to investigate possible side effects on tissues which are penetrated by the shockwaves on their way from the skin to the stone. By doing this, attention was paid not only to the focus zone where the highest energy is delivered but also to the surrounding area where lower energy is released. In consequence both destructive and regenerative effects were seen in bony tissues [3]. A dose-dependent effect was detected with high energy leading to more destructive effects and lower energy leading to more regenerative effects on the treated tissue [4–6]. In the early 1990s, extracorporeal shockwave effects on bone and soft tissues have led to indicating this treatment also for musculoskeletal disorders [4, 6]. Consequently, specific devices for musculoskeletal focused Extracorporeal Shock Wave Therapy (fESWT) were introduced into the market. These devices focus the shock waves to a point which is approximately 4–6 cm apart from the application to the skin. Compared with the urologic lithotripters which recommended immersion of the patient in a water bath, this first generation of orthopedic devices had reduced and adjustable energy release. Coupling to the patient's body was performed by ultrasound gel and aiming was realized by ultrasound [7]. In a consensus meeting in 1995, instructions were established for the use of extracorporeal shock waves in musculoskeletal indications [8]: (a) high energy only, (b) small “focus,” (c) anesthesia, (d) imaging guided application, (e) avoiding growth plates, (f) no acute injuries, (g) soft tissue pain in the proximity to bones (insertional tendinopathy), and (h) tendinopathies with extraosseous calcification.

In the early 2000s, devices featuring ballistic pressure waves were introduced into the Extracorporeal Shock Wave Therapy (ESWT) market. These waves are produced mechanically by a compressed air driven projectile which hits the applicator. This technology is since named radial ESWT (rESWT). The respective devices are much cheaper, smaller, and easier to handle. However, the maximum rESWT energy is delivered at the applicator to skin interface and focused shock waves peak pressure is about 100 times higher while the pulse duration is 1000 times shorter [9]. The clinical effect of rESWT could soon be demonstrated [10] and today rESWT is a widely accepted method with comparable results specifically for superficial musculoskeletal disorders [11, 12].

This review paper updates the current knowledge with respect to the historical paradigms as set in 1995 [8].

## 2. Materials and Methods

This narrative review presents eight different ESWT paradigms which were extracted from a historical German consensus meeting held in 1995. We evaluated if these paradigms are still true after 20 years of further development of the method.

Historically, most research related to musculoskeletal ESWT literature was published in German language and in books or journals which are not referenced in Medline. Therefore, a systematic search was judged not to be a reasonable approach.

The bases for the current investigation are the authors' databases, containing both historical Medline listed papers on ESWT and also historical ESWT articles which were published in German language. The content of these articles is further reported.

For each of the eight individual paradigms, the historical background is addressed. Developments over time and recent perspectives to these topics were analysed also from the authors' literature databases.

## 3. Results

### 3.1. High Energy Only?

Historically, the companies provided the users with different specifications of the used energy levels, some of them used the applied energy flux density (ED), and others used the voltage (kV) led into the device to produce the shock waves. In particular, the description of the voltage is device depending and therefore a comparison between different technologies (devices from different producers) is meaningless. So the convention was made to use ED (mJ/mm^2^) as the comparable parameter. It turned out that it is not enough to look at only one parameter. So it is no wonder that there are many conflicting publications due to the different energy descriptions [13, 14].

Beside the well-known shock wave effect of disintegration of concrements, a stimulation of fibrous tissue could be demonstrated to occur and this different biologic mechanism was dose-dependent [15].

Consequently and already in the early 1990s musculoskeletal ESWT was divided into “low” (0.08–0.23 mJ/mm^2^) and “medium” (14–18 kV) energy applications [8]. Not concordant with the former, a classification of low (<0.08 mJ/mm^2^), medium (0.08–0.28 mJ/mm^2^), and high (>0.28 mJ/mm^2^) energy was introduced and established [5, 16]. Evidence was obtained from an experimental study, demonstrating that “energy flux densities of over 0.28 mJ/mm should not be used clinically in the treatment of tendon disorders” [5]. Initially, low energy ESWT was called “pain therapy” and anesthesia was not considered a “conditio sine qua non” [8]. Early reports demonstrated promising results with low energy ESWT for soft tissue injuries like tennis elbow and plantar fasciitis [8]. Meanwhile, soft tissue indications were equally established for low energy fESWT and also rESWT. Comparable results are published regarding plantar fasciitis [17–19], Achilles [20, 21], and patellar tendinopathy [20, 22, 23]. A recent systematic review, respectively, confirms that “there is no scientific evidence in favor of either rESWT or fESWT with respect to treatment outcome” [24].

### 3.2. Small “Focus” Only?

Historically, ESWT was performed with lithotripters and also the first generation of musculoskeletal ESWT devices was based on the focused technology. Respectively, maximum energy was applied to a small area 5–10 cm below the applicator and this energy was concentrated in an area with a diameter of 5–10 mm [1]. Therefore, painful syndromes originating from a larger area were not considered as an indication for ESWT [6, 8]. Similarly, radiating or referred pain syndromes without a clear anatomic substrate were not regarded suitable for ESWT [6].

At that time, the fact that relevant energy is also measurable peripherally to the focal zone was neglected. Accepted indications were nonunion fractures, plantar fasciitis, tennis elbow, and calcific shoulder tendinopathy [8]. The “small focus only” statement was held until the invention of the radial technology [20], with the maximum energy delivered at the tip of the applicator. Due to the smaller sizes and lower costs of the devices, rESWT has increasingly been used all over the world. Even if the applied energy diminishes by square relative to the penetration depth, also this method has meanwhile clearly demonstrated its effectiveness for soft tissue injuries in level 1 studies [18, 19].

In a next step, rESWT was applied to treat more complex musculoskeletal symptoms associated with trigger points. The underlying mechanism of action is explained by the concept of myofascial pain [25]. Recently and inspired by traditional Chinese medicine, ESWT acupuncture has been invented [26].

### 3.3. (Local) Anesthesia?

Anesthesia allows applying shockwaves with higher intensities. Derived from kidney stone and nonunion fracture experience, high energy was proposed for orthopedic ESWT indications [8, 27]. Consequently, in the early 1990s it was suggested to adapt anesthesia (full, regional, or local) according to the applied energy level [8]. As a result of analgesia or anesthesia, several randomized controlled studies failed to demonstrate a significant advantage of ESWT against sham treatment [28, 29]. It was in 2005 when two randomized controlled studies revealed that local anesthesia at least reduces the effect of ESWT for plantar fasciitis [30, 31] and this effect was only partly compensated by applying higher energy levels under local anesthesia [31]. Comparable negative local anesthesia effects were demonstrated for insertional Achilles tendinopathy [32].

Nowadays, (local) anesthesia is still regarded as helpful for bone indications [33] but is not recommended for soft tissue ESWT [24].

Meanwhile, there is evidence from experimental research that the pain producing effect of ESWT is responsible for the release of neuropeptides (like substance P) initiating both central and local trophic effects to increase metabolism in bradytrophic tissues [24, 34]. It was experimentally demonstrated that “… ESWT dose-dependently activates and sensitizes primary afferent nociceptive C-fibers, and that both activation and sensitization were prevented if local anesthesia was applied” [34].

### 3.4. Imaging Guided Application?

**Figure 1:**
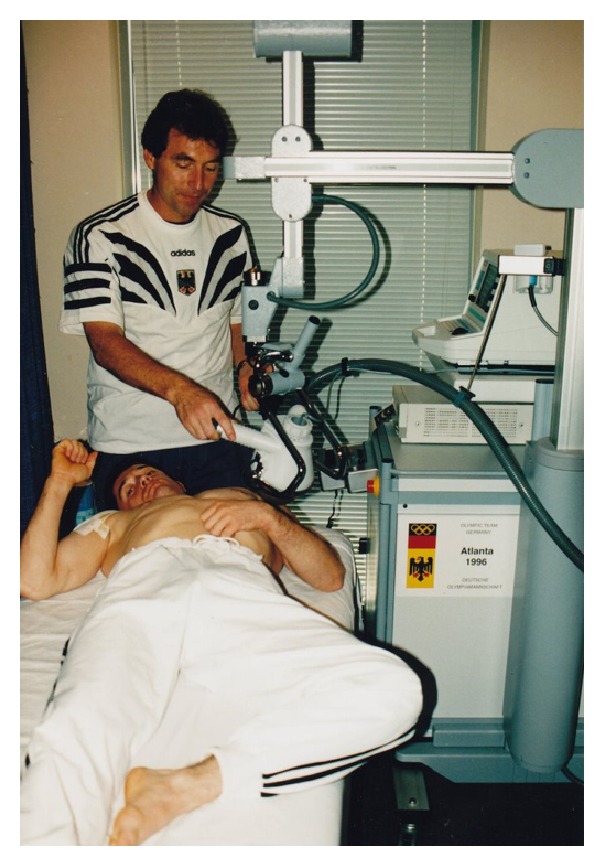
Initiation of the ESWT technology to treat Olympic athletes during the 1996 Olympic Games in Atlanta.

At the beginning of the orthopedic shock wave era, it was generally agreed that focal degenerative lesions within the injured tissues are responsible for the painful syndromes and should be exactly targeted by ESWT. Therefore, visualizing aiming devices were demanded [8]. Fluoroscopy was already integrated in all urologic fESWT devices which were used also for the initial years to treat orthopedic injuries. However, visualization of soft tissues was not possible. In 1995, in our center, the first fESWT device was installed to specifically treat sport orthopedic soft tissue indications. Most importantly, it was radiation free. An inline sonography system was incorporated in order to aim exactly the shock waves at the structure of interest. In 1996, this machine was available for the German team athletes during the Atlanta Olympic Games (Figure 1). Even if it was not stationary and its volume, weight, and price were considerably reduced compared with the lithotripters, transportation was a logistic effort. Therefore, really small and mobile ESWT devices were requested [8]. Again, urologists took this next step and applied the principle and the technology of an already existing device for intracorporeal ballistic lithotripsy to treat orthopedic soft tissue indications percutaneously.

That new technology produced pressure waves and not real shock waves, but the term radial shock wave was generally agreed upon and is used since [11]. Nowadays, ballistic devices have been developed with electromagnetic working mechanisms.

Users and investigators found out that aiming at the most painful area was sufficient or even superior to aiming just at an anatomically given landmark which was identified by imaging. This procedure has consequently been demonstrated to be superior and was termed “biofeedback” [35]. One well-known example is a double-blind, randomized placebo-controlled study on ultrasound-guided fESWT for plantar fasciitis [29].

Actually, focusing by biofeedback is also the cornerstone for myofascial trigger point ESWT [25]. However, the treatment of bone lesions like nonunions and osteochondrosis dissecans still needs image guided application, for example, by fluoroscopy.

### 3.5. Growth Plates?

In an experimental study on proximal rat tibiae, dysplastic lesions could be identified following high energy fESWT (20 kV, 1500 shock waves) [36]. As a consequence from this study, ESWT was regarded to be contraindicated in a juvenile population [6].

Only two years later, another animal study was published demonstrating no negative histological differences comparing fESWT effect with the untreated contralateral femoral head of immature rabbits [37]. Another experimental rabbit study was published in German language. The investigators applied 800 focused impulses (0.32 mJ/mm^2^) which is comparable to a high fESWT in a human bone application. Obviously, these two papers were underestimated in the scientific world [38]. For rESWT, a recently published rat experiment could detect “no negative effects” when 1500 or 3000 impulses of 4 bar were applied to the immature rat knees [39].

Even if initially mentioned anecdotally already in 1995 [40] it was only recently when the first clinical case series reported both safety and effectiveness when Osgood Schlatter or Sever's diseases were treated by using rESWT [41, 42].

### 3.6. Acute Injuries?

When introducing musculoskeletal ESWT, it was declared to be indicated for chronic injuries. The reason for this was that in general a new treatment modality should provide evidence before being spread out to the public, and, as long as the evidence is missing, it should be recommended only for patients, who already have been treated by other options. This means that three months of conservative treatment should have been performed without success before ESWT is indicated as an alternative to operative treatment [6, 8]. Extensive technical, manpower, and time requirements have been advocated as rationales for this limitation [8]. Additionally, economic factors limited the musculoskeletal ESWT application. Consequently, most research was traditionally made for conservatively pretreated injuries with a history of more than three months. International shock wave societies still consider only nonacute pathologies (http://www.digest-ev.de/leitlinien/). With the advent of cheaper and more flexible ESWT devices, this rule has been broken. For instance, in acute and operatively treated long bone fractures high energy fESWT effectively reduced the number of nonunions [43]. Contrary to this, in a randomized controlled study rESWT treatment was inferior to stretching for plantar fasciitis patients when patients were not pretreated and complained about symptoms under six weeks [44].

If ESWT can be relevant to effectively treat acute muscular or tendon strains is currently not known and respective research is needed.

### 3.7. Tendinopathies with Extraosseous Calcification

Historically, only mechanical (and not biologic) ESWT effects were regarded as relevant in medicine. At the transmission through tissues with similar acoustic properties (soft tissue) a minor amount of energy is released. It was assumed that the resulting mechanical effect is negligible. In contrast, high acoustic impedance differences exist between cortical bone (6.12 × 10^6^ kg/m^2^s) and soft tissue (e.g., muscle = 1.66 × 10^6^ kg/m^2^s). ESWT consequently releases a large amount of mechanical energy at the interface. This concept was the rationale not only to treat kidney stones but also to treat soft tissue calcifications [6]. Initially, a real destruction of bone was not detected as a result from ESWT [6], but later experimental research demonstrated a dose-dependent induction of cortical fractures and periosteal detachment [45]. Relevant acoustic impedance differences exist also at the interface between tendon and bone. Therefore, well-defined insertional tendinopathies like tennis elbow and plantar fasciitis were thought to be also eligible for ESWT treatment specifically when combined with a spur [8].

These treatment principles were held until the invention of the rESWT with a completely different technology. Historically, the main differences between fESWT and rESWT are as follows: (a) principle of generation = pneumatic rESWT versus electrohydraulic, piezoelectric, or electromagnetic fESWT, (b) wavelength = 0.15 to 1.5 m (rESWT) versus 1.5 mm (fESWT), and (c) maximum pressure = 1 (rESWT) versus 10–100 (fESWT) MPa and penetration depth = 2–5 cm (rESWT) versus 5–20 cm (fESWT) [9]. Nowadays, there are also ballistic devices available with electromagnetic working mechanisms accelerating the projectile to hit the applicator. Clinically most important thing is that the maximum energy in rESWT is delivered at the interface between the applicator head of the device and the skin and diminishes its energy inside the treated tissue by the square of the penetration depth [9].

As a result, rESWT was applied to tendon lesions, featured by their immediately subcutaneous localization and by a large area of injured tissue. Midportion Achilles tendinopathy and patellar tendinopathy fulfil these criteria and have been demonstrated to be an indication for rESWT [20, 46, 47]. Based on current evidence, we are unable to prefer fESWT or rESWT for musculoskeletal soft tissue injuries [11, 23]. Conflicting evidence exists from the results of two studies that directly compared fESWT to rESWT in plantar fasciitis and patellar tendinopathy patients [19, 23]. FESWT revealed moderately superior results compared to rESWT in plantar fasciitis, while no difference was demonstrated between the two applications regarding patellar tendinopathy [19, 23].

### 3.8. Three Sessions Only?

The number of required treatment sessions is a relevant parameter in principle. Recently, systematic research recommends “three treatment sessions at 1-week intervals, with 2000 impulses per session and the highest energy flux density the patient can tolerate” [24]. However, historical reports do not adequately address that detail [4, 8]. Analogue to and derived from the lithotripsy nonunion fractures have been treated with high energy predominantly in one session. The reason for this procedure is most probably based upon the intensive effort required by anesthesia and fluoroscopy. For the soft tissue conditions a wide range (1 to 10) of treatment sessions was initially instructed [4, 6]. The need of standardization of treatment regimens in randomized controlled trials established one to three ESWT sessions at weekly intervals as a standard clinical practice regardless of the underlying pathology [12, 17, 18, 23, 46, 48–53].

Recently, there have been a few reports which retrospectively addressed the number of rESWT sessions needed to treat soft tissue pathologies such as trigger digits, symptomatic calcified shoulder tendinopathy, and plantar fasciitis. These studies revealed that pretreatment symptom duration was significantly correlated with the number of rESWT sessions applied [54]. Additionally, there is evidence that there is a dose-related ESWT effect with lower energy flux densities [mJ/mm^2^] requiring more treatment sessions to obtain the same effect [55].

Discussion is still going on about which parameters or which combination of parameters should be used to maximize the effect of ESWT treatment for a specific indication. In this context, it has to be mentioned that comparability of studies should not be reduced on one single parameter (e.g., energy flux density).

In clinical practice, ESWT is rarely used as a monotherapy. Strategic loading and/or exercises are usually prescribed in addition to shock waves, a fact that in general RCTs have not adequately addressed. An individualized intervention should be considered depending initially on the type and characteristics of the pathology [56].

## 4. Discussion

The most important finding of this review is that all historical paradigms as set for musculoskeletal ESWT in 1995 did not withstand the technical and clinical developments over the last 20 years. The initial phase of the musculoskeletal ESWT was driven by side effect research in context with lithotripsy investigation and the first orthopedic applications have been performed by urologists [4]. Principles which were already known from more than two million lithotripsies in men and from respective animal studies were transferred and adapted to musculoskeletal indications.

At the beginning of the musculoskeletal shock wave age it was thought that the higher the energy is, the better the outcome would be. For soft tissue pathologies it was early realized that lower ESWT intensities are able to induce tissue regeneration instead of necrotic reactions [5]. The pain resulting from the ESWT is clearly depending on the energy intensity [34] but clinical focusing was shown to improve the treatment results especially when performed without local anesthesia [30, 34]. Specific ESWT devices for musculoskeletal conditions were produced. Further reduction of the applied energy was delivered with the rESWT technology. So and over the years, devices became much more flexible/mobile and had reduced volume, weight, and costs.

There are an increasing number of high quality ESWT studies for musculoskeletal conditions published in the literature. It can be summarized without exaggeration that ESWT is the best analyzed treatment modality in the orthopedic field. This statement includes also operative interventions. A recent systematic musculoskeletal ESWT review concludes that there is more need for high level studies [12]. But the question to be answered in future is not if ESWT works but rather which treatment protocol and parameters are the best for specific and well described conditions [47]. Research finally has to follow clinical practice, where treatment protocols are individualized.

Until now, clinical ESWT research is aiming exclusively at detecting the success of ESWT applied following a standardized protocol. The question, however, if ESWT is similarly effective in each stage of a given musculoskeletal indication is completely unanswered up to date. For instance, a “tendon pathology continuum model” has been described [56]. Derived from this, tendinopathy is “no longer a ‘one size fits all' diagnosis” [57]. It is to expect that different stages of a given pathology will respond differently to ESWT. Moreover, monotherapies are rarely used in clinical practice. Given the former, future randomized controlled work should focus on assessing and comparing more realistic treatment protocols.

## 5. Conclusion

With the exception of bone related conditions, modern musculoskeletal ESWT is performed with energy below 0.28 mJ/mm^2^ and without anesthesia. The size of the tissue area to be treated can be small or large. “Biofeedback” is superior to imaging guided focusing. ESWT application in apophyseal osteochondral lesions in patients with open growth plates seems to be promising and safe. ESWT protocols should be adapted to the stage and chronicity of the treated pathology.
